# Phytogenically Synthesized Zinc Oxide Nanoparticles (ZnO-NPs) Potentially Inhibit the Bacterial Pathogens: In Vitro Studies

**DOI:** 10.3390/toxics11050452

**Published:** 2023-05-10

**Authors:** Mo Ahamad Khan, Showkat Ahmad Lone, Mohammad Shahid, Mohammad Tarique Zeyad, Asad Syed, Aquib Ehtram, Abdallah M. Elgorban, Meenakshi Verma, Mohammad Danish

**Affiliations:** 1 Department of Microbiology, Jawahar Lal Nehru Medical College (JNMC), Aligarh Muslim University, Aligarh 202002, India; ahamadk065@gmail.com; 2 Department of Microbiology, Government Medical College, Baramulla 19310, India; 3 Department of Agricultural Microbiology, Faculty of Agriculture Science, Aligarh Muslim University, Aligarh 202002, India; mohd.zeyad@gmail.com; 4 Department of Botany and Microbiology, College of Science, King Saud University, P.O. Box 2455, Riyadh 11451, Saudi Arabia; assyed@ksu.edu.sa (A.S.); aelgorban@ksu.edu.sa (A.M.E.); 5 La Jolla Institute for Immunology, San Diego, CA 92037, USA; aehtram@Lji.org; 6 Centre of Research & Development, Department of Chemistry, Chandigarh University, Mohali 160055, India; 1707meenakshi@gmail.com; 7 Department of Botany, Faculty of Life Sciences, Aligarh Muslim University, Aligarh 202002, India; danish.botanica@gmail.com

**Keywords:** green synthesis, ZnO-NPs, MDR bacteria, anti-biofilm, virulence factors, EPS and membrane permeability

## Abstract

The usefulness of nanoparticles (NPs) in biological applications, such as nanomedicine, is becoming more widely acknowledged. Zinc oxide nanoparticles (ZnO-NPs) are a type of metal oxide nanoparticle with an extensive use in biomedicine. Here, ZnO-NPs were synthesized using *Cassia siamea* (L.) leaf extract and characterized using state-of-the-art techniques; UV–vis spectroscopy, XRD, FTIR, and SEM. At sub-minimum inhibitory concentration (MIC) levels, the ability of ZnO@*Cs*-NPs to suppress quorum-mediated virulence factors and biofilm formation against clinical MDR isolates (*Pseudomonas aeruginosa* PAO1 and *Chromobacterium violaceum* MCC-2290) was tested. The ½MIC of ZnO@*Cs*-NPs reduced violacein production by *C. violaceum*. Furthermore, ZnO@*Cs*-NPs sub-MIC significantly inhibited virulence factors such aspyoverdin, pyocyanin, elastase, exoprotease, rhamnolipid, and the swimming motility of *P. aeruginosa* PAO1 by 76.9, 49.0, 71.1, 53.3, 89.5, and 60%, respectively. Moreover, ZnO@*Cs*-NPs also showed wide anti-biofilm efficacy, inhibiting a maximum of 67 and 56% biofilms in *P. aeruginosa* and *C. violaceum*, respectively. In addition, ZnO@*Cs*-NPs suppressed extra polymeric substances (EPS) produced by isolates. Additionally, under confocal microscopy, propidium iodide-stained cells of *P. aeruginosa* and *C. violaceum* show ZnO@*Cs*-NP-induced impairment in membrane permeability, revealing strong anti-bacterial efficacy. This research demonstrates that newly synthesized ZnO@*Cs*-NPs demonstrate a strong efficacy against clinical isolates. In a nutshell, ZnO@*Cs*-NPs can be used as an alternative therapeutic agent for managing pathogenic infections.

## 1. Introduction

The widespread and indiscriminate use of antibacterial drugs/antibiotics has hastened the emergence of resistance to multiple drugs in pathogenic bacteria, and is a serious global problem [1]. Most of the blame for the establishment of multi-drug resistance (MDR) can be attributed to “selection pressure” brought on by the excessive and indiscriminate use of antibiotics for both medicinal and non-medical purposes [2]. Multi-drug resistance in pathogenic microorganisms around the world results in a significant number of fatalities and hospitalizations each year [3]. A 2019 study estimated that the number of death associated with bacterial AMR as 4.95 million, with more than 75% of them attributed to six leading pathogens which includes *Escherichia coli*, followed by *Staphylococcus aureus, Klebsiella pneumoniae, Streptococcus pneumoniae, Acinetobacter baumannii*, and *Pseudomonas aeruginosa* [4]. Many pathogenic bacteria have a mechanism for communication called quorum sensing (QS), which controls the synthesis of virulence proteins and the development of biofilms [5,6]. Bacteria produce signals called autoinducers (AIs), which are used by cells to communicate with one another [7]. AIs have been identified in both Gram-positive and Gram -negative bacteria as oligopeptides and N-acyl-homoserine lactones (AHLs), respectively [8]. There are three main QS systems in *Pseudomonas aeruginosa*: lasI/R, rhlI/R, and pqsA/R. These systems are connected by AIs such as oxododecanoyl homoserine lactone (C12-HSL), and butyryl homoserine lactone (C4-HSL), and *Pseudomonas* quinolone-based intracellular signal (PQS) produced by *Pseudomonas* spp. [9]. AIs activate genes that control the synthesis of virulence factors such as pyocyanin, pyoverdin, hemolysins, elastase, and proteases [10]. By interfering with bacterial cell–cell communication mechanisms, quorum quenchers (QQ) are compounds that prevent the synthesis of several virulence factors, such as proteases, toxins, adhesins, siderophores, swarming motility, and biofilm formation [11,12]. Hence, more effective antibacterial alternatives with unique action mechanisms are urgently needed.

Current developments in nanotechnology, notably the capacity to prepare highly organized nano-particulates of any size and shape, have opened avenues the formation of novel biocidal compounds [13]. Nanoparticles (NPs) are referred to as “a wonder of modern medicine” [14]. NPs with significant antimicrobial properties have proven to be promising alternatives for the treatment of multi-drug resistant bacteria [15]. Nanomaterials are gaining a lot of interest because of their unique properties, which include a lot of useful traits for biological applications [16]. Due to their different physicochemical and biological features over their bulk phase, NPs have been extensively studied as antibacterial agents in medical and biological areas such as medical diagnostics, biosensors, and personal care products [17,18]. It is stated that antibiotics kill perhaps a half dozen different disease-causing organisms but nano-materials can kill some 650 cells [19]. Due to their unique catalytic, optical, electrical, magnetic, antibacterial, wound-healing, and anti-inflammatory capabilities, metal oxide nanoparticles (MONPs) have been intensively explored [20]. Recent research demonstrates that the cytotoxicity of various NPs, notably MNPs, can cause prokaryotic cell growth suppression as well as cell death in eukaryotic cells [21].

Zinc oxide nanoparticle is one of the MONPs that is very intriguing since it has many diverse uses in areas such as the optical, piezoelectric, magnetic, and gas-sensing fields [22]. In addition to these characteristics, ZnO-NPs have excellent catalytic efficiency, good adsorption ability, and are increasingly used in the production of sunscreens [23,24]. In recent years, advances in nanotechnology have led to an increase in use of NPs in the medical field and as a therapy for infectious diseases [25]. It has been found that MONPs such as ZnO and silver have superior effectiveness against antibiotic-resistant bacteria [26]. Recently, it was found that ZnO-NPs function as anti-quorum sensing molecules for the laboratory strain *Pseudomonas aeruginosa* PAO1, reducing the production of several virulence factors such as pyocyanin, PQS signal, pyochelin, and haemolytic activity without having a major growth inhibitory effect [27]. Likewise, bio-fabricated ZnO-NPs, synthesized using *Parthenium hysterophorus* L. leaf extract, showed a size-dependent antifungal efficacy against plant fungal pathogens [28]. Jayaseelan and colleagues [29] described a low-cost, straightforward process for creating ZnO-NPs by a unique microbiological approach using *Aeromonas hydrophila* as an environmentally benign reducing and capping agent, and they investigated their efficacy against harmful bacteria and fungi. Employing *Aloe barbadensis* miller leaf extract, ZnO-NPs were synthesized and showed a strong antibacterial activity against pathogenic microbes [30]. In addition, the cytotoxic and genotoxic effects of ZnO-NPs are reported. For example, studies conducted by Feng et al. (2017) show that the concentration and particle size of ZnO-NPs negatively affect the gut microbiota. The microflora of ileal digesta and blood plasma were changed, and *Lactobacillus* was reduced by increasing concentrations of ZnO-NPs.

*Cassia siamea* (L.), also known as *Senna siamea* (L.), (commonly known as Kassod tree) belongs to the subfamily Caesalpinoideae of the leguminous family. It is native to South and Southeast Asia. In Asian countries, people have utilized its many parts in a wide range of ways, both medicinally and in daily life. For indigestion and as an expectorant, a mixture of the root, leaf, and flower extracts is used. The antioxidant (Kaur et al., 2006), anti-inflammatory, and analgesic activities/properties of *C. saimea* are well known [31,32]. Additionally, the leaf and flower extract of this plant show antibacterial/antimicrobial properties against several clinical isolates [33,34,35]. Using different plant organs (leaf and flowers), nanomaterials were synthesized and showed antibacterial efficacy. Given the numerous remarkable benefits and extensive utility of ZnO-NPs, and medicinal importance of *C. siamea* (L.), a thorough and meticulous systematic effort was made to accomplish the following goals: (i) employing leaf extract from *Cassia siamea* (L.) to synthesize ZnO-NPs in a green manner and its characterization; (ii) determination of the minimum inhibitory concentration (MIC) and minimum bactericidal concentration (MBC) of ZnO-NPs against bacterial pathogens *Pseudomonas aeruginosa* PAO1 and *Chromobacterium violaceum* MCC 2290; (iii) evaluation of ZnO-NPs potential for quorum sensing and biofilm inhibition as well as their potential involvement in suppressing synthesis of quorum-sensing controlled virulence factors and pathogenesis in *Pseudomonas aeruginosa* PAO1 using different microbiological assays; (iv) assessment of phytogenically synthesized ZnO-NPs on extra polymeric substances (EPS) and cellular permeability of bacterial pathogens.

## 2. Materials and Methods

### 2.1. Bacterial Strain and Media Used

The standard strains of bacterial pathogens used, *Pseudomonas aeruginosa* PAO1 and *Chromobacterium violaceum* MCC 2290 (Pune, India), in this study were obtained from the Department of Microbiology, Jawahar Lal Nehru Medical College (JNMC), Aligarh Muslim University (AMU), Aligarh, India. Bacterial cultures were grown and maintained on/in Luria–Bertani (LB) medium at 25 °C for 24 h and maintained at 4 °C in a refrigerator. The cultures utilized in this investigation were grown on the same nutritional medium.

### 2.2. Details of Instrument and Specifications 

Transmission electron microscopy (TEM) was carried out using JOEL-2100, Tokyo, Japan at 200 kV. Scanning electron microscopic (SEM) analysis was performed using JSM 6510LV (JEOL, Tokyo, Japan). UV spectra of synthesized ZnO-NPs were taken by using UV–vis spectrophotometer-UV5704S from Electronics, India Ltd. X-ray diffraction (XRD) was performed using Mini Flex™ II XRD system, Rigaku Corporation, Tokyo, Japan. Fourier-transform infra-red spectroscopy (FTIR) analysis was performed on PerkinElmer FTIR spectrometer Spectrum Two (Perkin Elmer Life and Analytical Sciences, Shelton, CT, USA).

### 2.3. Green Synthesis of Zinc Oxide Nanoparticles (ZnO-NPs)

*Cassia siamea* (L.) aqueous extract was obtained by dissolving 10 g of dried leaf powder in 100 mL of double-distilled water (DDW). The *C. siamea* (L.) leaves were washed and dried before aqueous extract preparation. When the leaves were completely dry, they were ground into a moderately fine powder. After that, 10 g of the leaf powder was mixed with 100 mL of de-ionized water in glass containers and stirred for 3 h. After stirring, the mixture was placed in a water bath (at 60 °C) for 1 h. Finally, the extracts were filtered using Whatman filter paper-1 and stored at 4 °C for further use. ZnO-NPs were made by combining 1.0 mM zinc nitrate (ZnNO_3_) hexahydrate with an aqueous solution in a ratio of 9:1. The reaction mixture was heated for 3–4 h until the cream-colored precipitate was obtained. This cream-colored precipitate was centrifuged for 15 min at 3000 rpm, and the resultant pellet was collected on a glass plate. Pellet was oven-dried for 2–3 days at 45 °C. The powder was then finely ground using a metallic mortar–pestle when it had dried completely.

### 2.4. Characterization of ZnO-NPs

#### 2.4.1. UV–Visible Spectroscopy

The green synthesized ZnO-NPs were preliminary characterized using a UV–visible spectrophotometer. The synthesis of ZnO-NPs was monitored by recording the UV–visible spectra of solutions after one hour intervals until no changes in absorbance were found. The absorbance spectra were captured in quartz cuvettes with a 1 cm path length between 250 and 800 nm at room temperature. To adjust the background absorption, double-distilled water was utilized as a reference [36].

#### 2.4.2. X-ray Diffraction

The ZnO-NPs obtained were characterized in powdered form by an X-ray diffractometer. The diffraction pattern was acquired by CuK_α_ radiation (=1.54 Å) at 30 mA current and operating at 40 kV. The angle of direction (2θ) data were recorded in the range of 20°–80°. Average crystalline size was calculated by Debye–Scherrer’s equation:

D = Kλ/βcosθ

where D is the crystallite size in nm, λ is the X-ray wavelength, β is the full width at half maximum, and θ is the Bragg’s angle of reflection [37].

#### 2.4.3. Fourier-Transform Infrared Spectroscopy (FTIR)

The transmittance spectra were recorded by placing the dried powder of ZnO nanoparticles to spectroscopic grade KBr (mass ratio of about 1:100). FTIR analysis was performed at 4 cm^−1^ resolutions in diffuse reflectance mode in KBr pellets [38].

#### 2.4.4. Scanning Electron Microscopy and EDX

Scanning electron micrographs of ZnO-NPs were obtained using SEM equipped with EDX spectrometer to carry out analysis of constituting elements. The electron beams were accelerated at 15 kV. Images were obtained at 2500–35,000× magnification [39].

#### 2.4.5. Transmission Electron Microscopy (TEM)

For TEM analysis, aqueous suspension of ZnO-NPs was made in double-distilled water followed by sonication at 30% amplitude for 15 min. About 10 μL of the suspension was transferred to TEM grid for analysis and excess amount of suspension was removed by soft filter paper. The grid was then allowed to dry at 80 °C for 6  h. Imaging was performed at 200  kV in the magnification range of 300,000–100,000× magnification.

### 2.5. Agar Well Diffusion Assay

The agar well diffusion assay was used to assess the preliminary antibacterial activity of green synthesized ZnO-NPs [40,41]. (see Supplementary Section S2.5 for detailed description). 

### 2.6. Minimum Inhibitory Concentration (MIC) and Minimum Bactericidal Concentration (MBC) 

The lowest concentration at which bacterial growth is inhibited is known as an antibacterial agent’s minimum inhibitory concentration (MIC). The MIC and MBC values ZnO-NPs were determined following the method as previously described by Alekish et al. [42] (see Supplementary Section S2.6 for detailed description). 

### 2.7. ZnO@Cs-NP-induced Inhibition of Virulence Factors 

#### 2.7.1. Extraction and Quantification of Pyocyanin 

The previously published technique was used to extract the pyocyanin from pathogenic isolate *Pseudomonas aeruginosa* by culturing the cells in pseudomonas broth medium [43]. In Pseudomonas broth (PB) medium supplemented with varying doses (0–20 μg mL^−1^) of ZnO@*Cs*-NPs, *P. aeruginosa* was cultivated for 18 h. Chloroform was used to remove 5 mL of culture supernatant from overnight growth (3.0 mL). Re-extraction of the organic phase in 0.2 N HCl (hydrochloric acid) was performed (1.2 mL). At 520 nm, the absorbance of the pink aqueous phase was recorded.

#### 2.7.2. Evaluation of Pyoverdine

The previously published procedure was used to evaluate the pyoverdine pigment [44]. In essence, the culture of *P. aeruginosa* was centrifuged to obtain the cell-free supernatant. The increasing doses (0–20 μg mL^−1^) of ZnO@*Cs*-NPs were used to grow the cells of *P. aeruginosa*, and the control group received no treatment. At 460 nm, the fluorescence emission intensity was measured after 0.1 mL of cell-free supernatant and 0.9 mL of 50 mM Tris-HCl buffer (pH 7.4) were combined. The samples were read at 400 nm using UV–visible spectrophotometer. 

#### 2.7.3. Exo-Protease Activity Assessment 

As previously mentioned by Whooley et al. [45], the azocasein degradation assay was used to measure the exo-protease activity. In a nutshell, *P. aeruginosa* was grown in media supplemented with increasing concentrations (0–20 μg mL^−1^) of ZnO@*Cs*-NPs. A control set (no any treatment of NPs) was kept for comparison. By centrifuging, cell-free supernatant was recovered. A total of 0.3% azocasein was combined with 100 μL of supernatant. A solution of 0.05 M Tris-HCl, pH 7.5, and 0.5 mM CaCl_2_ was used to produce the azocasein. After 15 min of incubation at 37 °C, the reaction was stopped by adding 500 μL of 10% *w*/*v* trichloroacetic acid (TCA). The optical density (OD) of the supernatant was measured at 400 nm after the sample had been centrifuged for 10 min at 12,000 rpm.

#### 2.7.4. Elastase Activity Evaluation

The previously published procedure of Li et al. [46] was utilized to measure the elastolytic activity using elastin Congo red (ECR). (See Supplementary Section S2.7.4 for detailed description). 

#### 2.7.5. Assessment of Rhamnolipid 

As previously mentioned by Laabei et al. [47], the orcinol technique was used to assess the effect of green synthesized ZnO-NPs on the production of rhamnolipid [48]. In 0.6 mL of diethyl ether, 300 microliters of cell-free supernatant from *P. aeruginosa* was extracted, then the organic phase was separated. The orcinol (0.19% orcinol in 53% sulphuric acid) was added to each sample after the organic phase had been dried by evaporation at 37 °C and reconstituted in 0.1 mL of deionized water. After being heated for 30 min at 80 °C, the sample was cooled to room temperature. At 421 nm, the absorbance of the samples was recorded. 

#### 2.7.6. Swarming Motility

Using the previously reported procedure [49], bacterial movement on agar soft plates was inhibited. In a nutshell, different concentrations of ZnO@*Cs*-NPs were amended in 0.5% LB agar plates for swarming motility. The plates were spotted with mL of an overnight frown culture, which was then incubated for 18 h. The diameter of swarm was recorded in mm. 

#### 2.7.7. Extraction and Quantification of Violacein

The culture of *C. violaceum* was grown in LB medium for 18 h in the absence and presence of varying concentrations of ZnO@*Cs*-NPs. To precipitate the insoluble pigment, 1.0 mL of overnight grown culture from each treatment group was centrifuged at 10,000 rpm for 5 min. Later, the pellet was re-suspended in 1.0 mL of DMSO and then vortexed vigorously for 30 sec. Following that, the solution was centrifuged again to remove the bacterial cell debris. The optical density (OD) of colored supernatant was recorded at 585 nm using UV-2600 Spectrophotometer, Shimadzu, Japan [50].

### 2.8. Anti-Biofilm Activities 

#### 2.8.1. Biofilm Inhibition Assay

The inhibiting potential of green synthesized ZnO-NPs (ZnO@*Cs*-NPs) to initial cell adhesion was tested using the biofilm inhibition assay reported by O’Toole [51]. (See Supplementary Section S2.8.1 for detailed description). 

#### 2.8.2. Light Microscopy of Biofilm 

Biofilms were grown on glass coverings in the presence and absence of ZnO nanoparticles. To remove the planktonic cells, the cover slips were washed with sterile phosphate buffer and stained with 0.1% crystal violet (CV). The attached cells and biofilms were visualized under light micros at 40× magnification [52].

### 2.9. Haemolytic Activity

Haemolytic analysis was performed as previously described by Saghalliet al. [53]. Initially, 5% blood agar (BA) plates were made with increasing concentration of ZnO@*Cs*-NPs, however, control plates were not amended with NPs. Wells were punched in the plates, and gaps were filled with soft agar. Then, 100 μL of overnight grown culture of *P. aeruginosa* PAO1 was poured into wells and plates were incubated at 37 °C for 24 h. After incubation, the zone of clearance on blood agar plates was detected and recorded.

### 2.10. Time Dependent ZnO@Cs-NP-Induced Growth-Inhibition Assay and Colony Forming Ability (CFU Count)

By measuring the optical density (OD at 620 nm) of ZnO@*Cs*-NP-treated and untreated bacteria using 96 well microtiter plates, the impact of NPs on the development of *P. aeruginosa* and *C. violaceum* isolates was evaluated [54] See Supplementary Section S2.10 for detailed description).

The bacterial cultures (1 × 10^8^ CFU mL^−1^) were extracted, treated with increasing doses of ZnO@*Cs*-NPs, and used to determine the viable cell count. To determine the CFU, 100 μL bacterial suspensions of treated and untreated cells were spread out on Luria agar (LA) plates [55]. Colonies were enumerated following incubation (at 37 °C. for 24 h) and the results were displayed as a function of the concentration of ZnO@*Cs*-NPs.

### 2.11. Permeability Determination by Confocal Laser Scanning Microscopy (CLSM)

CLSM was used to assess the cell membrane permeability of clinical isolates (*P. aeruginosa* and *C. violaceum*) following the protocols described in earlier research [55,56,57,58]. In order to perform this, cells with damaged cell membranes were found using the probe PI. Biofilms that had not been treated and those that had been treated with 10 μg mL^−1^ of ZnO@*Cs*-NPs were both stained with 100 μM PI for 15 min at room temperature in the dark after three sterile phosphate buffer washes. After being incubated with PI, cells were put on a microscope slide, three times rinsed with PBS, and then observed using CLSM.

### 2.12. Inhibition of Exopolysaccharides (EPS) 

Exopolysaccharide (EPS) levels in control and ZnO@*Cs*-NP-treated cultures were measured using the usual methodology with some changes [59,60,61]. In a nutshell, the tested bacterial cultures were cultured at 37 °C for 24 h in the presence of various doses (0–20 μg mL^−1^) of ZnO@*Cs*-NPs. No treatments were administered to the control group. Bacterial cultures were centrifuged when the incubation period was completed in order to extract the cell-free supernatant. The culture supernatant was mixed with cold ethanol that had been diluted by a factor of 3:1 before being incubated at 4 °C for an overnight period to precipitate the EPS. The amount of EPS was calculated and the sugars were estimated using the Dubois method.

### 2.13. Statistical Analysis 

There were three duplicates of each experiment. The information presented in this study is averaged data with a plus or minus standard deviation. The *t*-test was used to compare the control group to the treated group. *p* values below 0.05 were considered significant.

## 3. Results and Discussion

### 3.1. UV–VIS Spectrophotometer, XRD, FTIR, SEM-EDX, and TEM Analysis of ZnO-NP

**Figure 1:**
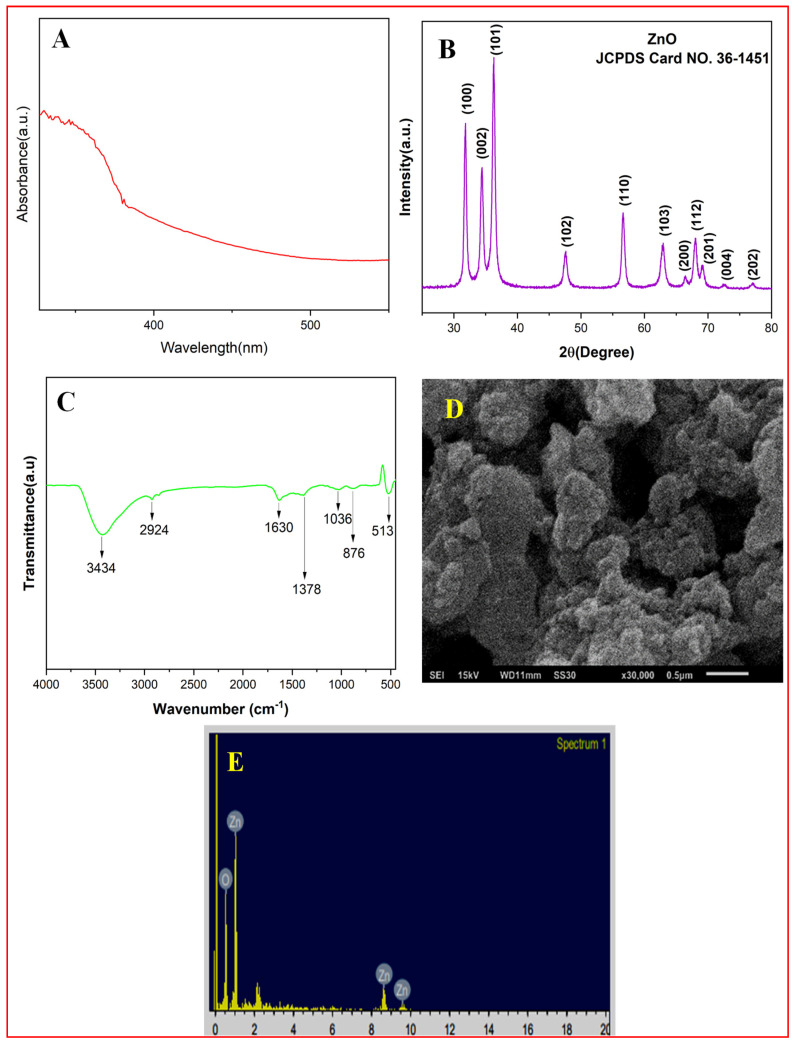
Characterization of phytogenically synthesized ZnO-NPs: (**A**) UV–vis absorption spectrum of ZnO nanoparticles of *Cassia siamea* (L.) leaf extract; (**B**) XRD patterns of ZnO nanoparticles; (**C**) FT-IR spectrum of synthesized ZnO nanoparticles; (**D**) the SEM micrograph of ZnO nanoparticles at 35,000× magnification; and (**E**) EDX pattern.

A UV–visible spectrophotometer was used to perform preliminary characterization of the synthesized white ZnO nanoparticles in the range of 200 nm and 600 nm. The absorption peak at 384 nm indicates the formation of ZnO nanoparticles (Figure 1A). Similar to the current investigation, various works identified and characterized the green synthesized ZnO-NPs using the extracts of diverse plant samples such as seed, leaf, stems, etc. [62,63,64,65]. UV–vis data suggest the formation of phases of ZnO NPs. The band-gap energy (E) of ZnO-NPs was computed using Planck’s equation:

E = hc/λ

where h is Planck’s constant, c is light velocity, and λ is the wavelength. The E (band-gap energy) for ZnO-NPs is found to be 3.326 eV, which is coherent with previous reports.

Furthermore, in order to characterize the ZnO-NPs, the dried powder was subjected to XRD analysis. The Cu-kα (1.54 Å) was employed as radiation source for X-ray diffraction study of the synthesized ZnO nanoparticles, in the diffraction angle ranging from 20º to 80°. Figure 1B depicts the different diffraction peaks (100), 002), (101), (102), (110), (103), (200), (112), (201), (004), and (202) at diffraction angles (31.80), (34.36), (36.39), (47.62), (56.72), (62.98), (66.51), (68.09), (69.22), (72.55), and (76.91), respectively. These peaks are in accordance with JCPDS card number (36–1451) and establishes the formation of pure phase ZnO-NPs (space group P63 mc). The Debye–Scherrer formula given as

D = Kλ β ∗ cos θ

was used for the estimation of the average crystallite size~13nm, where D is size of the ZnO-NPs; β is full width of XRD peak at the half maximum; λ is wavelength (1.5406 Å) of radiation, and K is the Debye–Scherrer constant.

Similar to the current investigation, numerous phytogenically/greenly synthesized ZnO-NPs were characterized using the XRD analysis [66,67,68].

Fourier-transform infrared spectroscopy (FTIR) is a significant instrument for the characterization of green synthesized nanoparticles because it gives valuable information about the rotational and vibrational modes of motion of molecules [69]. Purified ZnO-NPs were mixed with 10 mg potassium bromide (KBr) powder and dried to remove moisture content to make the dry powder sample of ZnO-NPs. Infrared spectra were recorded in the region of 400–4000 cm^−1^ at room temperature (Figure 1C). Peaks at 1036 and 1378 cm^−1^ are due to bending vibration of C-O and phenolic O-H groups, respectively. The peak at 513 cm^−1^ shows the Zn–O stretching vibrations [70]. The negligible peak at 2924 cm^−1^ corresponds to alkanes C-H. The intense peak at 3434 cm^−1^ is due to O-H stretching and a small peak at 1630 cm^−1^ corresponds to the presence of primary amines. The presence of these organic biomolecules aids in reduction and facilitates stabilization of the synthesized ZnO nanoparticles.

One of the methods for determining the form of nanoparticles that is most frequently employed is scanning electron microscopy (SEM). In order to depict the size, shape, and surface morphology of green synthesized ZnO-NPs, SEM and TEM analyses were performed. The form of ZnO nanoparticles, which range from spherical to oval to spheroidal, was seen, supporting the TEM findings. The surface morphology and size of ZnO-NPs were analyzed using scanning electron microscopy (SEM). Individual ZnO-NPs and aggregates were visible in the SEM image. As a result of being obtained from the powdered form of ZnO-NPs, SEM pictures can be viewed as enormous clusters of particles. There has been prior documentation of variation in the size and form of green synthesized ZnO nanoparticles. The reason why there is such variance in the form of green manufactured nanoparticles is because some of the particles are capped and stabilized at a smaller size, while other particles are stabilized at a larger size. Figure 1D shows the scanning electron micrograph (SEM) of ZnO-NPs at 15,000× and 35,000× magnifications at 15 and 20 kV, respectively. The surface scanning makes it clear that spherical and oval-shaped nanoparticles are the most common shapes discovered. Despite SEM not being able to measure particle size, it is still possible to see that the nanoparticles are about 100 nm. Figure 1E shows EDX pattern in which the highest elemental weight percent detected is of zinc, with 62.0%.

### 3.2. Anti-Bacterial Activity of Cassia siamea (L.)-Synthesized ZnO Nanoparticles (ZnO@Cs-NPs)

**Figure 2:**
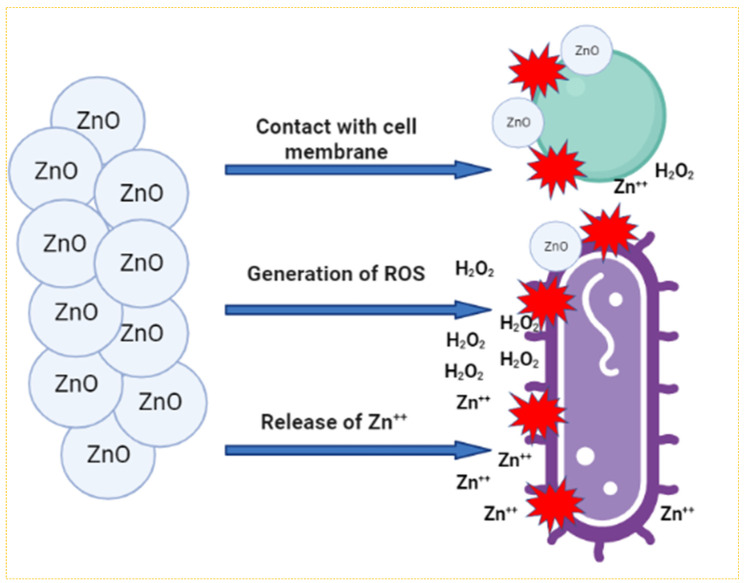
Different possible schematic representation showing the mechanisms of antibacterial effectiveness of ZnO-NPs against the bacterial pathogens, including ROS formation, Zn^2+^ release, internalization of ZnO-NPs into bacteria, and electrostatic interactions.

The agar well diffusion experiment was performed to examine the antibacterial activity of phytogenically synthesized ZnO@*Cs*-NPs against pathogenic isolates *P. aeruginosa* PAO1 and *C. violaceum*. With a minimum inhibition zone of 15 mm against ZnO@*Cs*-NPs, *C. violaceum* is discovered to be the most resistant of the studied bacteria. *P. aeruginosa* PAO1 is found to exhibit maximum zones of inhibition of 20 mm for ZnO@*Cs*-NPs (Table 1). The larger surface area of ZnO@*Cs*-NPs, which allows for more surface contact with microbes, is thought to be the cause of their increased antibacterial activity. The synergistic interaction between particles and natural chemicals is a significant factor in the literature-documented increased antibacterial activity of ZnO@*Cs*-NPs. Similarly, phenazine-1-carboxamide and NPs were discovered to work in concert to increase the antibacterial action against MRSA strains by a factor of 32 while also generating morphological changes to the bacterial cell wall. The antibacterial activity of green synthesized ZnO@*Cs*-NPs works by targeting the respiratory chain and cell division, which eventually results in cell death, further improving their bactericidal effect. Protection against therapeutic agents including NPs is one of the main purposes of the bacterial cell walls and membrane. Bacteria are categorized as a result of the variations in their cell wall architectures. There are essentially two layers of lipopolysaccharides in the Gram-negative cell wall, also known as the cell envelope. Gram-positive cells, in contrast, have thicker cell walls that are predominantly made of peptidoglycans, a single type of molecule. It has been reported that suspensions of ZnO-NPs produce an enhanced level of reactive oxygen species (ROS). Such reactive species are superoxide anion (O^−2^), hydrogen peroxide (H_2_O_2_), and hydroxide (OH^−^). This ROS formation is the main mechanism responsible for ZnO-NPs antibacterial activity (Figure 2). Similarly, *Phoenix roebelenii* palm-leaves-synthesized ZnO-NPs showed a considerable antibacterial activity against Gram-negative (*Salmonella typhi* and *Escherichia coli*) and Gram-positive (*Staphylococcus aureus* and *Streptococcus pneumoniae*) bacterial pathogens [71]. Furthermore, Imade and workers [72] used agar well diffusion and broth dilution assays to measure the antibacterial activity of ZnO-NPs and their effectiveness against pathogenic microorganisms of *Salmonella enterica*, *Klebsiella pneumoniae*, *Bacillus cereus* MTCC 430, and *Staphylococcus aureus* 26923. Other works also analyzed the antibacterial effectiveness of phytogenically synthesized NPs [73].

### 3.3. Minimum Inhibitory Concentration (MIC) and Minimum Bactericidal Concentration (MBC) 

MIC values of ZnO@*Cs*-NPs against *Pseudomonas aeruginosa* PAO1 and *C. violaceum* are 80 and 60 µg mL^−1^, respectively, with MBC values of 160 and 120 µg mL^−1^ (Table 2). Similar to our study, Zn-NPs show antibacterial activity against poultry-associated foodborne pathogens *Salmonella* spp., *Escherichia coli*, and *Staphylococcus aureus* with 80, 60, and 30 µg mL^−1^ MIC and 160, 140, and 100 µg mL^−1^ MBC, respectively [74]. Sub-MIC concentrations were used to evaluate the reduction in biofilm and QS-mediated virulence factors. Likewise, in another study, a comparable antibacterial activity was observed for *Carumcopticum* L. (ajwain) extract-loaded manganese ferrite (MnFe_2_O_4_) NPs against bacterial pathogens such as *Staphylococcus aureus*, *Pseudomonas aeruginosa*, *Staphylococcus epidermitis*, *Bacillus subtilis*, *E. coli,* and methicillin-resistant Staphylococcus aureus (MRSA) [75]. Moreover, silver nanoparticles (Ag-NPs) have been linked to a number of additional biological functions, including antioxidant, anticancer, and anticoagulant properties [76]. The relevant sub-MIC level was used to evaluate the suppression of biofilms and QS-mediated virulence factors.

### 3.4. Inhibition of Virulence Factors in P. aeruginosa PAO1

**Figure 3:**
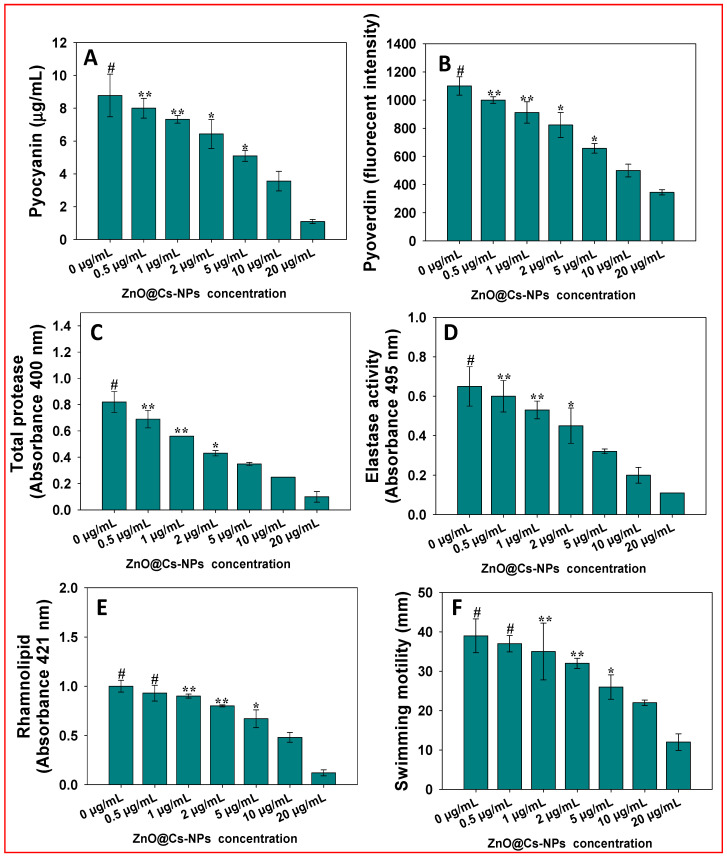
Impact of varying concentrations of green synthesized ZnO-NPs on virulence factors of *Pseudomonas aeruginosa* PAO1: pyocyanin (**A**), pyoverdine (**B**), total protease (**C**), elastase activity (**D**), rhamnolipid (**E**), and swimming motility (**F**). Data are represented as percentage of biofilm inhibition. In these figures, the histograms represent the mean value of biofilm percentage over the untreated control (100%), while error bars show standard deviation (SD, n = 3) with a significance of * *p* ≤ 0.05, ** *p* ≤ 0.005, and # *p* ≤ 0.001. The symbols *, **, and # represent the significant difference between treatment with untreated control (i.e., 0 µg mL^−1^).

*Pseudomonas aeruginosa* PAO1 is recognized for its virulence component pyocyanin, which is a blue–green pigment. Pyocyanin concentrations were observed to be 7.02 g mL^−1^ in untreated culture of *P. aeruginosa*. The pyocyanin of *P. aeruginosa* is inhibited after being treated with varying rates of ZnO@*Cs*-NPs in a dose-dependent manner. For instance, at 0.5, 1, 2, 5, 10, and 20 μg ZnO@*Cs*-NPs mL^−1^, there is a 11.10, 23, 26, 36, and 58%, reduction in pyocyanin, respectively (Figure 3A). The pathogen *P. aeruginosa* becomes more harmful due to pyocyanin, which also disrupts a number of cellular processes. Pyocyanin and phenazine-1-carboxylic acid, its precursor, have been shown to affect the immunological modulatory protein expression and hinder the human respiratory cilia’s ability to beat properly in cystic fibrosis patients [77]. It has been discovered that pyocyanin relates to the severity of disease and is known to cause oxidative stress [78]. Pyocyanin promotes the growth of biofilms and suppresses the host’s defense mechanism by causing human neutrophils to apoptosis at higher rates. Furthermore, pyocyanin is known to cause oxidative stress, and its presence has been linked to disease severity [79]. Pyocyanin promotes biofilm formation and suppresses the host defense mechanism, resulting in increased apoptosis rates in human neutrophils [80].

The luminous siderophore pyoverdine, another part of *P. aeruginosa* pathogenicity, serves a crucial role in the infection of the host. ZnO@*Cs*-NPs reduce the production of pyoverdine in a concentration-dependent manner, similar to other variables. Pyoverdine production is maximally (*p* ≤ 0.001) reduced by 49% when using growth media supplemented with 2 μg ZnO@*Cs*-NPs mL^−1^ compared to untreated control (Figure 3B). More than 50% of pyoverdine’s activity is inhibited after exposure to 2 μg ZnO@*Cs*-NPsmL^−1^. By preventing the identification of lipocalin linked to neutrophil gelatinase, pyoverdine aids in the formation of *P. aeruginosa* infection, particularly in the lungs of people suffering from cystic fibrosis (CF) [81]. As pyoverdine competes with mammalian transferrin for iron (Fe), it also results in an iron deficit in the tissues of the host [82].

By using an exo-protease assay that breaks down the azocasein, protease synthesis was evaluated. Exo-protease activity of pathogenic isolate *P. aeruginosa* that degrades the azocaseinis greatly and significantly (*p* ≤ 0.001) reduced by 71.18% when treated with 20 μg ZnO@*Cs*-NPs mL^−1^ (Figure 3C). The increased bacterial invasion results from the proteases’ ability to circumvent the host’s defense mechanisms and cleave the host cell proteins. The outcome demonstrates that ZnO@*Cs*-NPs has the string ability to block the exo-protease activity. In the past, Qais et al. (2021) discovered that exo-protease activity in *P. aeruginosa* was suppressed by 86% when treated with greenly synthesized silver nanoparticles (Ag-NPs) [83].

The potential of ZnO@*Cs*-NPs to suppress the elastase activity (that breaks down elastin in a QS-dependent manner) was also investigated. The ZnO@*Cs*-NPs exert a dose-dependent influence on *P. aeruginosa*’s elastase activity. For example, ZnO@*Cs*-NPs at 20 μg mL^−1^ maximally inhibit the *LasB* elastase activity of isolate by 53.32% compared to the untreated control (Figure 3D). The development of biofilms and the QS-controlled virulent phenotype are both critically dependent on the production of las proteins [84]. It is known that *P. aeruginosa* produces a number of hydrolytic enzymes, including elastases, which are responsible for the breakdown of tissue components and ultimately interfere with the immune-system-regulating mechanisms of the host. 

The rhamnolipids are glycolipidic biosurfactants that have now been discovered in a number of pathogenic bacterial species [85,86]. The treatment of various doses of ZnO@*Cs*-NPs shows a significant decrease in the formation of rhamnolipid, similar to other virulence factors. There is a 60% reduction in rhamnolipid production in the presence of 2 μg ZnO@*Cs*-NPs mL^−1^ (Figure 3E). Rhamnolipids cause the QS-controlled swimming motility and the dissemination of biofilms at the infection site [87]. As it is necessary for the formation of biofilms, their dissemination, chemotaxis, and pathogenicity, motility plays a significant role in bacterial life. Some molecular motors propel the flagella, enabling the bacteria to swim. At all evaluated concentrations of ZnO@*Cs*-NPs, the swimming motility of *P. aeruginosa* PAO1 is significantly reduced (*p* ≤ 0.001) in a dose-related manner (Figure 3F). *P. aeruginosa* culture that had not been treated swam to the edge of the plate, having a swim diameter of 89.3 mm.

### 3.5. Inhibition of Violacein

**Figure 4:**
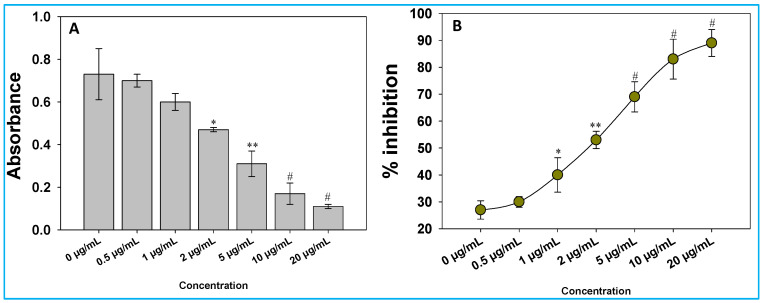
Qualitative analysis of violacein inhibition in *C. violaceum* in the absence and presence of ZnO@*Cs*-NPs; absorbance of violacein production (**A**) and NPs-dependent inhibition (**B**). Data represent the mean value of three independent replicates (n = 3), while error bars show standard deviation (SD, n = 3) with a significance of * *p* ≤ 0.05, ** *p* ≤ 0.005, and # *p* ≤ 0.001. The symbols *, **, and # represent the significant difference between treatment with untreated control (i.e., 0 µg mL^−1^).

The effectiveness of green synthesized ZnO@*Cs*-NPs to inhibit the violacein produced by *C. violaceum* was evaluated both qualitatively as well quantitatively (which was measured by spectrophotometrically). The increasing concentrations (0–20 µg mL^−1^) of ZnO@*Cs*-NPs show a significant reduction in violacein production. The maximum reduction is recorded at higher concentration of ZnO@*Cs*-NPs. For instance, in the presence of 20 μg ZnO@*Cs*-NPs mL^−1^, pigment synthesis is reduced by 89% compared to untreated control (Figure 4). The *C. violaceum* produces violacein pigment under direct control of the bacterial QS system (CviR) through the C6-HSL signalling molecule, so any reduction in pigment production is a clear indication of impairment in the QS architecture [88]. The findings suggest the inhibitory effect of green synthesized ZnO@*Cs*-NPs on *C. violaceum* QS-mediated function.

### 3.6. Biofilm Formation

**Figure 5:**
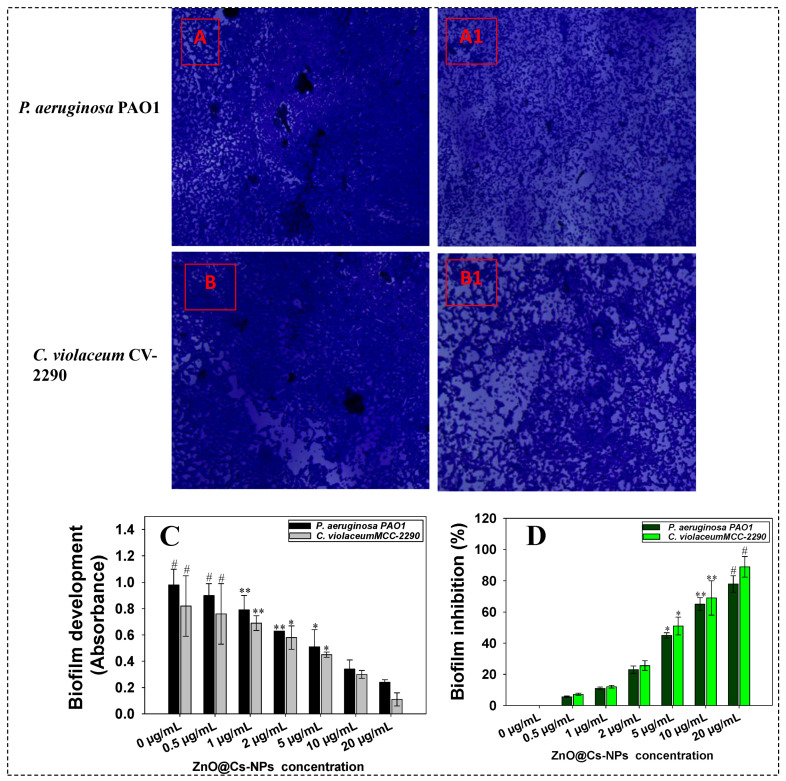
Impact of different concentrations of green synthesized ZnO-NPs on biofilm formation by test bacterial pathogens: Light microscopic images of untreated and ZnO-NPs treated biofilms formed by *Pseudomonas aeruginosa* PAO1; untreated (0 µg mL^−1^) control (**A**), treated with 5 µg mL^−1^ (**A1**). Panel **B** denotes the biofilm formed by *C. violaceium* under ZnO@*Cs*-NPs stress; untreated (0 µg mL^−1^) control (**B**), treated with 5 µg mL^−1^ (**B1**). Panel **C** and **D** represent the measurement of biofilm inhibition as quantified by crystal violet (CV) staining; development of biofilm by bacterial pathogens in the presence of increasing concentrations ZnO@*Cs*-NPs (**C**). Percent inhibition in biofilm formation (**D**). In these figures, the histograms represent the mean value of three independent replicates (n = 3), while error bars show standard deviation (SD, n = 3) with a significance of * *p* ≤ 0.05, ** *p* ≤ 0.005, and # *p* ≤ 0.001. The symbols *, ** and # represent the significant difference between treatment with untreated control (i.e., 0 µg mL^−1^).

Biofilms are bacterial communities that attach to the surfaces in a self-produced polymeric substance. The ability of ZnO@*Cs*-NPs to stop the pathogenic isolates from forming biofilm was examined in the current investigation. A ZnO@*Cs*-NPs concentration-dependent decrease in biofilm formation of *P. aeruginosa* PAO1 (Figure 5A,A1) *C. violaceum* (Figure 5B,B1) was observed under light microscopy. Furthermore, the biofilm formed by pathogenic bacteria were quantitatively estimated. While calculating the biofilms developed by bacterial cells under ZnO-NPs stressed condition, a dose-dependent decrease in biofilm formation is recorded. For instance, when cells of *P. aeruginosa* PAO1 are treated with 0.5, 1, 2, 5, and 10 µg ZnO@*Cs*-NPs mL^−1^, the production of biofilm by the PAO1 strain is reduced by 11.07, 31.78, 50, and 68.2%, respectively (Figure 5C). Likewise, when *C. violaceum* biofilm is exposed to 20 μg ZnO@*Cs*-NPs mL^−1^, the maximum reduction in biofilm is 62%. Similarly, the % inhibition in bacterial biofilms was calculated (Figure 5D). These results indicate that the higher concentration of ZnO@*Cs*-NPs causes the maximum inhibitory effect on bacterial properties among the test concentrations. Microscopic examinations on glass coverslips confirm the quantitative results of biofilm inhibition obtained by microtiter plate assay. The untreated cells of the test pathogens produce a thick mat-like structure on the glass surface, as shown in the light microscopic picture. Biofilms have medicinal significance because of their role in bacterial pathogenesis and the difficulty of eradicating them using antibiotics [89]. Biofilms are thought to be responsible for over 80% of human infections [90]. The development and structural integrity of bacterial biofilms are influenced by autoinducer-based QS systems, which play a crucial role in pathogenicity [91]. 

### 3.7. Anti-Haemolytic Activity

We investigated how green synthesized ZnO@*Cs*-NPs affected the ability of *P. aeruginosa* to produce/synthesize extracellular hemolysin (phospho-lipase C), which results in hemolysis. By administering different dosages of ZnO@*Cs*-NPs to the bacterial cultures, the hemolysin production of *P. aeruginosa* PAO1 was evaluated both qualitatively and quantitively. For qualitative evaluation, 5% blood agar plates treated with increasing concentrations of ZnO@*Cs*-NPs were prepared. It is intriguing to note that *P. aeruginosa* hemolyzed human red blood cells, and ZnO nanoparticles dose-dependently prevent this. The results reveal a considerably higher inhibition of hemolysis in a concentration-dependent manner (Figure S1A). Hemolysins cause the host cell lysis. Several hemolysins, such as phospholipase and lecithinase, work together to break down the lipids and lecithin [92]. These proteins also aid the invasion by generating the cytotoxic effects on host cells [93].

### 3.8. Inhibition of Colony-Forming Ability (CFU Counts)

**Figure 6:**
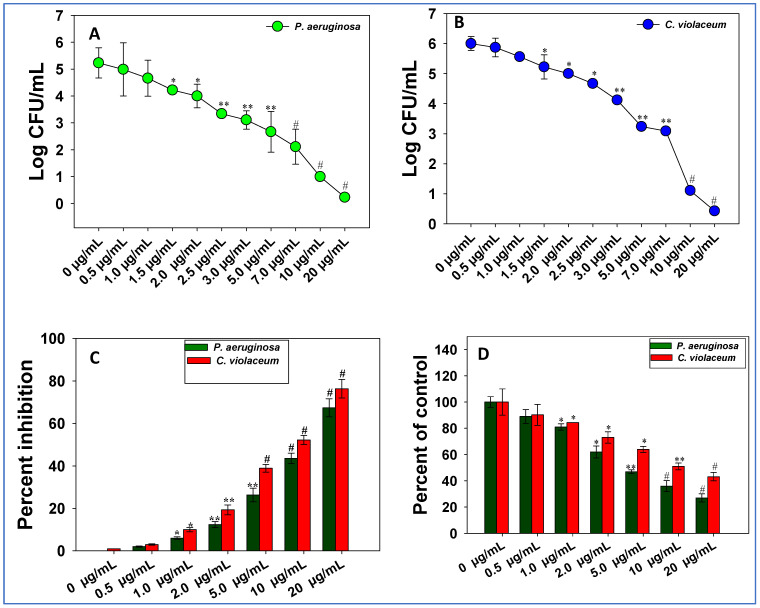
Impact of different concentrations (0−20 μg mL^−1^) of ZnO-NPs on CFU count of *P. aeruginosa* PAO1 (**A**) and *C. violaceum* (**B**). Panels (**C**,**D**) depicts the inhibition in extra polymeric substances (EPS) of bacterial pathogens. Bar diagrams represent the mean values of three independent replicates s (n = 3). Corresponding error bars represent the standard deviation (SD) of three replicates (SD, n = 3). In these figures, the histograms represent the mean value of three independent replicates (n = 3), while error bars show standard deviation (SD, n = 3) with a significance of * *p* ≤ 0.05, ** *p* ≤ 0.005, and # *p* ≤ 0.001. The symbols *, **, and # represent the significant difference between treatment with untreated control (i.e., 0 µg mL^−1^).

In order to determine the impact of ZnO@*Cs*-NPs on viable cell count of bacterial pathogens, 100 μL bacterial suspensions of treated and untreated cells were spread out on Luria agar (LA) plates and CFU was recorded. It was observed that the number of colony-forming counts (CFU mL^−1^) of *P. aeruginosa* PAO1 (Figure 6A) and *C. violaceum* (Figure 6B) decrease consistently with increasing concentrations of ZnO@*Cs*-NPs. The lower concentrations of NPs pose a lesser effect, and the higher concentration has the maximum notable effect on bacterial viability. For instance, concentrations of 20 μg ZnO@*Cs*-NPs mL^−1^ show the maximum inhibitory effect and highly reduce the cell viability of both bacterial pathogens. As a result, the growth inhibition reported in this study might be related to metal ion transport/uptake across membranes. The loss of bacterial cell respiration is one of the most common causes of bacterial cell viability decrease. The interaction of NPs with components of the bacterial plasma membrane results in respiratory inhibition, resulting in a reduction in cell viability. Similar to this, the increasing concentration of the chemical compound is reported to decrease the cellular permeability (in terms of CFU count mL^−1^) of bacterial cells [52].

### 3.9. EPS Inhibition

High-molecular-weight (HMW) natural polymers called extracellular polymeric substances (EPS) serve as the biofilms’ structural support [94]. The extracellular polymeric material is, therefore, the essential part of biofilms and also controls their physicochemical characteristics [95,96]. EPS makes up the majority of extracellular polymeric compounds’ components. The EPS acts as a barrier to protect bacterial cells by preventing chemotherapeutic substances such as antibiotics from penetrating the cells [97]. Due to the changed biofilm architecture brought on by the increased production of EPS, antimicrobial medicines are more resistant to these substances [98]. Targeting EPS production is also being studied as an alternative target for biofilm suppression because there is a positive association between EPS secretion and biofilm formation. ZnO@*Cs*-NPs successfully reduce the formation of EPS by test bacteria. For example, the 20 μg ZnO@*Cs*-NPs mL^−1^ significantly inhibits the EPS synthesized by *P. aeruginosa* and *C. violaceum* by 67% and 76%, respectively, over untreated control (Figure 6C,D).

EPS plays a significant role in the architecture of the biological system. In addition to shielding bacterial cells from drugs and environmental challenges, it gives the biofilms structural stability [99]. Targeting EPS is, therefore, thought of as an alternative technique to limit biofilms, because decreased EPS secretion is anticipated to have negative impacts on the capacity of bacterial pathogens to build biofilms [100]. Similarly, *Carum-copticum*-synthesized titanium dioxide nanoparticles (TiO_2_-NPs) significantly inhibit the EPS secreted by pathogenic isolates *P. aeruginosa* PAO1 and *E. coli* ATCC 25922 [101]. Furthermore, a dose-dependent inhibition of the EPS of clinical isolates *P. aeruginosa*, *S. aureus*, and *E. coli* was observed when bacterial cultures were treated with green synthesized ZnO-NPs [102].

### 3.10. Cellular Permeability of Bacterial Pathogens

**Figure 7:**
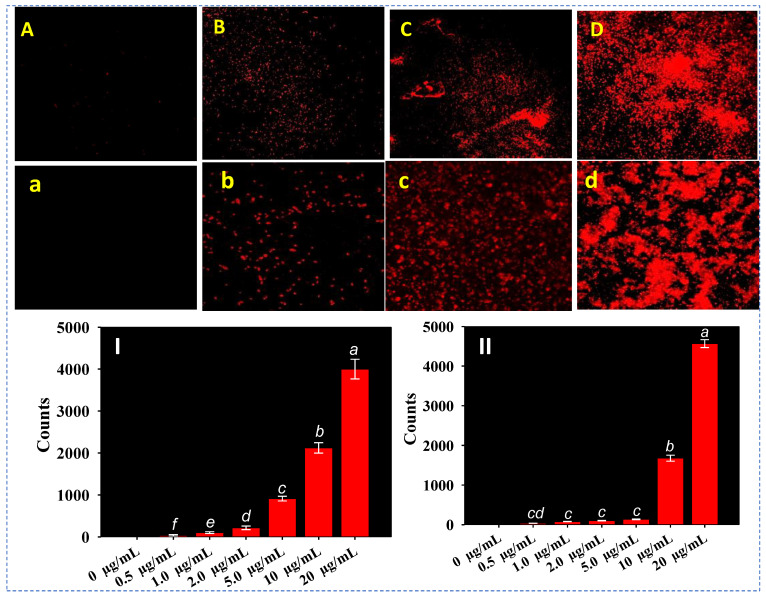
PI-stained and ZnO@*Cs*-NP-treated and untreated control confocal laser scanning microscopic (CLSM) images of *P. aeruginosa* PAO1; untreated control (**A**), treated with 5 µg mL^−1^ (**B**), 10 µg mL^−1^ (**C**), and 20 µg mL^−1^ (**D**) of ZnO@*Cs*-NPs and *C. violaceum*; untreated control (**a**), treated with 5 µg mL^−1^ (**b**), 10 µg mL^−1^ (**c**), and 20 µg mL^−1^ (**d**) of ZnO@*Cs*-NPs. Panels show that increasing concentrations of ZnO@*Cs*-NPs causes a considerable increase in the number of red-colored rod-shaped dead cells. Panels **I** and **II** show the cellular viability quantification of *P*. *aeruginosa* PAO1 (panel **I**) and *C. violaceum* (panel **II**). Bar diagrams represent the mean values of three independent replicates s (n = 3). Corresponding error bars represent the standard deviation (SD) of three replicates (SD, n = 3).

Propidium iodide (PI), a fluorescently labelled probe, was used to conduct the qualitative and quantitative evaluations of cell permeability in both tested bacterial pathogens. The increasing concentrations of ZnO@*Cs*-NPs, cause a significant decrease in cellular permeability of *P. aeruginosa* PAO1 (Figure 7A–D) and *C. violaceum* (Figure 7a–d), and increase the number of dead cells, observed under CLSM. Since PI stains the dead cells, therefore, metabolically inert cells of bacterial isolates show red rods on a black background following the excitation at 532 nm (λ_exc_). The red fluorescence of PI is not increased in untreated cells or increased somewhat. In quantitative estimation, a 5-6-fold increase in membrane-compromised cells of clinical isolates is recorded when cells of *P. aeruginosa* (Figure 7I) and *C. violaceum* (Figure 7II) are treated with 20 μgmL^−1^ of ZnO@*Cs*-NPs. Both the metal oxide and the type of bacterium are used to determine how reactive the nanoparticle is with bacterial cells. Additionally, the stability, concentration, and particle size of the NPs all affect their antibacterial effectiveness. A greater reaction between the NPs and the pathogens exists because the outer cellular membrane of certain bacterial strains has nanoscale holes. The bacterial cell membrane is subsequently damaged by the ROS, and the cells absorb PI. The antibacterial properties of ZnO@*Cs*-NPs are mostly due to the production of ROS such as OH^−^, H_2_O_2_, and O^2−^. Superoxide and hydroxyl radicals cannot enter bacterial cells because of their negative charges and must remain in contact with the bacteria’s outer surface [91]. H_2_O_2_, on the other hand, can enter bacterial cells [92]. According to this investigation into the synthesis and bactericidal potential, ZnO@*Cs*-NPs have effective antibacterial properties and can be utilized at the right dose to treat certain bacteria or affected tissue while minimizing side effects. The OH^−^ and H^+^ are formed when the holes divide water molecules. Similar to our investigation, as-synthesized copper oxide nanoparticles (CuNPs) caused oxidative stress and impaired the membrane permeability of multi-drug resistance pathogenic strains *Proteus vulgaris* (MTCC no. 426) and *Escherichia coli* (MTCC no. 739) [93].

## 4. Conclusions

The global rise in multidrug resistance has prompted scientists to focus on environmentally benign nanoparticle (NPs) production methods with effective anti-virulence effects. Using leaf extract of *Cassia siamea* (L.), zincoxide nanoparticles (ZnO@*Cs*-NPs) were synthesized and characterized. ZnO@*Cs*-NPs were discovered to effectively block the QS-controlled virulence factors of tested bacterial pathogens. The chemicals may inhibit the QS systems such as *LasR*-*RhlR*, *LasILasR*, and *PQS-MvfR*, which would explain their anti-virulence properties. According to research, putative mechanisms of ZnO-NPs interference include reduction in AHL synthesis, disruption of AHL binding to receptor proteins, antagonistic behavior toward regulatory proteins, and disruption of pilus formation. Additionally, greensynthesized ZnO@*Cs*-NPs successfully inhibit the growth of biofilm. The ability of Zn ions to enter the bacterial biofilm matrix accounts for the antibiofilm mechanism of action of ZnO@*Cs*-NPs. Therefore, the work reveals the potential of phytogenically synthesized ZnO@*Cs*-NPs to cure diabetic foot ulcers and topical disorders connected with pathogenic bacteria by inhibiting the QS and biofilms. However, it might also be used to coat the surface of medical implants to prevent bacterial adhesion and the bacterial illness that goes along with it. To pinpoint the mechanism behind antibacterial action, more study is required. Additionally, molecular investigations are needed to comprehend the precise mechanisms underlying anti-virulence tactics. It is recommended that the method of green synthesis can be applied for additional biological applications, and investigations at the gene level may be a better option for comprehending the interaction between NPs and gene expression.

## Figures and Tables

**Table 1:** Antibacterial activity (agar well diffusion assay) of green synthesized ZnO@*Cs*-NPs against bacterial pathogens.

Bacterial Pathogens	Zone of Inhibition (mm)
*P. aeruginosa* PAO1	20 ± 2.5
*C. violaceum* MCC 2290	15 ± 1.0

Values are the mean (mean ± S. D) of three (n = 3) independent replicates.

**Table 2:** MIC and MBC values of green synthesized ZnO@*Cs*-NPs against bacterial pathogens.

Bacterial Pathogens	Value (µg mL^−1^)	
	MIC	MBC
*P. aeruginosa* PAO1	60 ± 4.2	160 ± 5.8
*C. violaceum* MCC 2290	80 ± 3.1	120 ± 4.7

Values are the mean (mean ± S. D) of three (n = 3) independent replicates.

## Data Availability

The data presented in this study are confidential.

## Supplementary Materials

The following are available online at https://www.mdpi.com/article/10.3390/toxics11050452/s1, Figure S1: Impact of varying concentrations (0, 1, 2, 5 and 10 µgmL^−1^) of green synthesized ZnO-NPs on qualitative estimation of hemolycin produced by Pseudomonas aeruginosa PAO1 (Panel A).

## Abbreviations

MDR	Multi-drug resistance
XRD	X-ray diffraction
UV	Ultra-violet
FTIR	Fourier-transform infrared spectroscopy
SEM	Scanning electron microscope
TEM	Transmission electron microscope
MIC	Minimum inhibitory concentrations
MBC	Minimum bactericidal concentration
ZnO	Zinc oxide
NPs	Nanoparticles
CLSM	Confocal laser scanning microscope
EPS	Extra polymeric substances
CDC	Centres for Disease Control
AIs	Autoinducers
AHLs	N-acyl-homoserine lactones
QQ	Quorum quenchers
MONPs	Metal oxide nanoparticles
DDW	Double-distilled water
KBr	Potassium bromide
OD	Optical density
CaCl_2_	Calcium chloride
TCA	Trichloroacetic acid
LB	Luria–Bertani
CV	Crystal violet
CFU	Colony-forming unit
PI	Propidium iodide
QS	Quorum sensing
MRSA	Methicillin-resistant Staphylococcus aureus
