# Supplementary material for: Phytogenically Synthesized Zinc Oxide Nanoparticles (ZnO-NPs) Potentially Inhibit the Bacterial Pathogens: In Vitro Studies
Source: Toxics. 2023 May 10;11(5):452. doi: 10.3390/toxics11050452 (PMC10221642; doi:10.3390/toxics11050452)
Supplement: Supplementary file 1 [file toxics-11-00452-s001.zip › toxics-2362401-supplementary.pdf]

Article

Phytogenically synthesized Zinc oxide nanoparticles (ZnO-NPs) potentially inhibits the bacterial pathogens: *In vitro* studies

Mo Ahamad Khan[1], Showkat Ahmad Lone[2,†*], Mohammad Shahid[3,†,*], Mohammad Tarique Zeyad[3], Asad Syed[4], Aquib Ehtram[5], Abdallah M. Elgorban[4], Meenakshi Verma[6] and Mohammad Danish[7]

[1] Department of Microbiology, Jawahar Lal Nehru Medical College (JNMC), Aligarh Muslim University, Aligarh-202002, India;

[2] Department of Microbiology, Government Medical College Baramulla, Jammu and Kashmir-19310, India; showkat.microbio@gmail.com

[3] Department of Agricultural Microbiology, Faculty of Agriculture Science, Aligarh Muslim University, Aligarh-202002, India; shahidfaiz5@gmail.com (M.S), mohd.zeyad@gmail.com (M.T.Z)

[4] Department of Botany and Microbiology, College of Science, King Saud University, :P.O. 2455, Riyadh, 11451, Saudi Arabia; assyed@ksu.edu.sa (A.S), aelgorban@ksu.edu.sa (A.M.E)

[5] La Jolla Institute for Immunology, California, United States; aehtram@Lji.org (A.E)

[6] University Centre of Research & Development, Department of Chemistry, Chandigarh University, Gharuan, Mohali, India; 1707meenakshi@gmail.com

[7] Department of Botany, Faculty of Life Sciences, Aligarh Muslim University, Aligarh-202002, India; danish.botanica@gmail.com

* Correspondence: showkat.microbio@gmail.com (S.A.L) shahidfaiz5@gmail.com (M.S)

† These authors have contributed equally to this work and shared second authorship.

Material and methods

S2.5. Agar Well Diffusion Assay

The agar well diffusion assay was used to assess the preliminary antibacterial activity of green synthesized ZnO-NPs (Manandhar et al., 2019; Farjana et al., 2014). Both test bacterial pathogens were cultured in nutrient broth (NB) for overnight period to produce 10^6 colony-forming units (CFU) per millilitre. One-hundred microlitres of each bacteria culture was spread on the LB agar plates. With the use of sterilised micropipette tips, 8°mm agar wells were punched, and they were then filled with double-distilled water and ZnO-NPs. After that, the plates were incubated for 18 h at 37° C, and millimetre (mm) measurements of the zone of inhibition were made.

S2.6. Minimum Inhibitory Concentration (MIC) and Minimum Bactericidal Concentration (MBC)

The lowest concentration at which bacterial growth is inhibited is known as an antibacterial agent's minimum inhibitory concentration (MIC). The MIC and MBC values ZnO-NPs were determined following the method as previously described by Alekish et al. [30]. In a 96-well microtiter plate using 2,3,5-triphenyl tetrazolium chloride (TTC), the MIC of green synthesized ZnO-NPs against bacterial pathogens (*Pseudomonas aeruginosa* PAO1 and *Chromobacterium violaceum*) was determined. The bacterial culture was grown until it reached a McFarland standard of 0.5. The bacterial culture (10 μL) was then pipetted into wells containing 140 μL of nutritious broth with varying concentrations of ZnO-NPs (0 to 200 μg mL^{-1}). Nutrient broth (NB) without ZnO-NPs was taken as a control. The culture plate was incubated for 24 h at 37 °C. Following that, each well was loaded with 10 μL of TTC dye solution at a concentration of 20 mg mL^{-1} and incubated at 37 °C for 1 h. The MIC was determined by the concentration at which no change in colour was observed. Meanwhile, the MBC is the lowest antimicrobial concentration that kills bacteria while inhibiting their growth. Sub-culturing the well suspension from the MIC result onto nutrient agar gave the MBC. In brief, ten microliters of bacterial culture were put on an agar plate and incubated at 37ºC for 24 h. The MBC was defined as the lowest concentration that showed no bacterial growth. The tests were conducted in triplicate (n = 3).

S2.7.4. Elastase activity evaluation

The previously published procedure of Li et al. [34] was utilised to measure the elastolytic activity using elastin Congo Red (ECR). For the assay, 100 microlitre cell free supernatant of *P. aeruginosa* was added to 0.9 mL ECR buffer (5 mg mL^{-1} ECR and 1 mM $CaCl_2$ in 100 mM Tris, pH 7.5). For 3 h, the reaction mixture was incubated at 37 °C while being shaken. After adding 1.0 mL of 100 mM sodium phosphate buffer (pH 6.0) to the reaction to stop it, the sample was placed on ice for 30 min. Following a centrifugation step to remove insoluble ECR, the sample was measured for absorbance at 495 nm.

S2.8. Anti-biofilm Activities

S2.8.1. Biofilm Inhibition Assay

The inhibiting potential of green synthesized ZnO-NPs (ZnO@*Cs*-NPs) to initial cell adhesion was tested using the biofilm inhibition assay reported by O'Toole [39]. A 96-well microtiter plate filled with 180 μL of LB broth was used to evaluate the biofilm inhibitory activity of green synthesized ZnO-NPs. A 10 μL inoculum of *P. aeruginosa* (OD_{560}=1.0) and *C. violaceum* was pipetted into the individual well and incubated for 6 h at 37°C at static condition. Following incubation, a series (0, 0.5, 1.0, 2.0, 5.0, 10 and 20 μg mL^{-1}) of ZnO@*Cs*-NPs concentrations were introduced to the wells and incubated for 24 h at 37 °C in a static condition. After incubation, the cultures were removed, and wells were washed three times with PBS (Phosphate Buffer Saline) to eliminate the free-floating cells. The wells were stained with crystal violet dye (0.1% w/v) for 15 min and washed once again to remove any remaining free dye. Finally, the stained biofilms were de-stained with ethanol (95%) and left to rest for 30 min and measured the biofilm's OD at 620 nm using a microtiter plate reader.

Untreated wells were used as a control. The test was performed in triplicate (n =3). Biofilm inhibition percentage (%) was determined using the following formula.

$$\text{Biofilm inhibition (\%)} = (OD_{control} - OD_{treatment})/OD_{control} \times 100$$

S2.10. Time dependent ZnO@Cs-NPs-induced growth inhibition assay and colony forming ability (CFU count)

By measuring the optical density (OD at 620 nm) of ZnO@*Cs*-NPs-treated and untreated bacteria using 96 well microtiter plates, the impact of NPs on the development of *P. aeruginosa* and *C. violaceum* isolates was evaluated. Following the addition of ZnO@*Cs*-NPs to wells containing 200 µL of LB to achieve 10–50 µg mL^{-1}, both the isolates were inoculated at a rate of 1%. Cells that weren't treated with ZnO@*Cs*-NPs served as the standard. In order to prevent the variations caused by ZnO@*Cs*-NPs as a result of reflectance, additional controls of each concentration of ZnO@*Cs*-NPs were performed in parallel. After incubating the culture at 37 ^{0}C (for 24 h), microplate reader was used to detect absorbance (at 620 nm) at regular intervals of 2 h [42].

Results

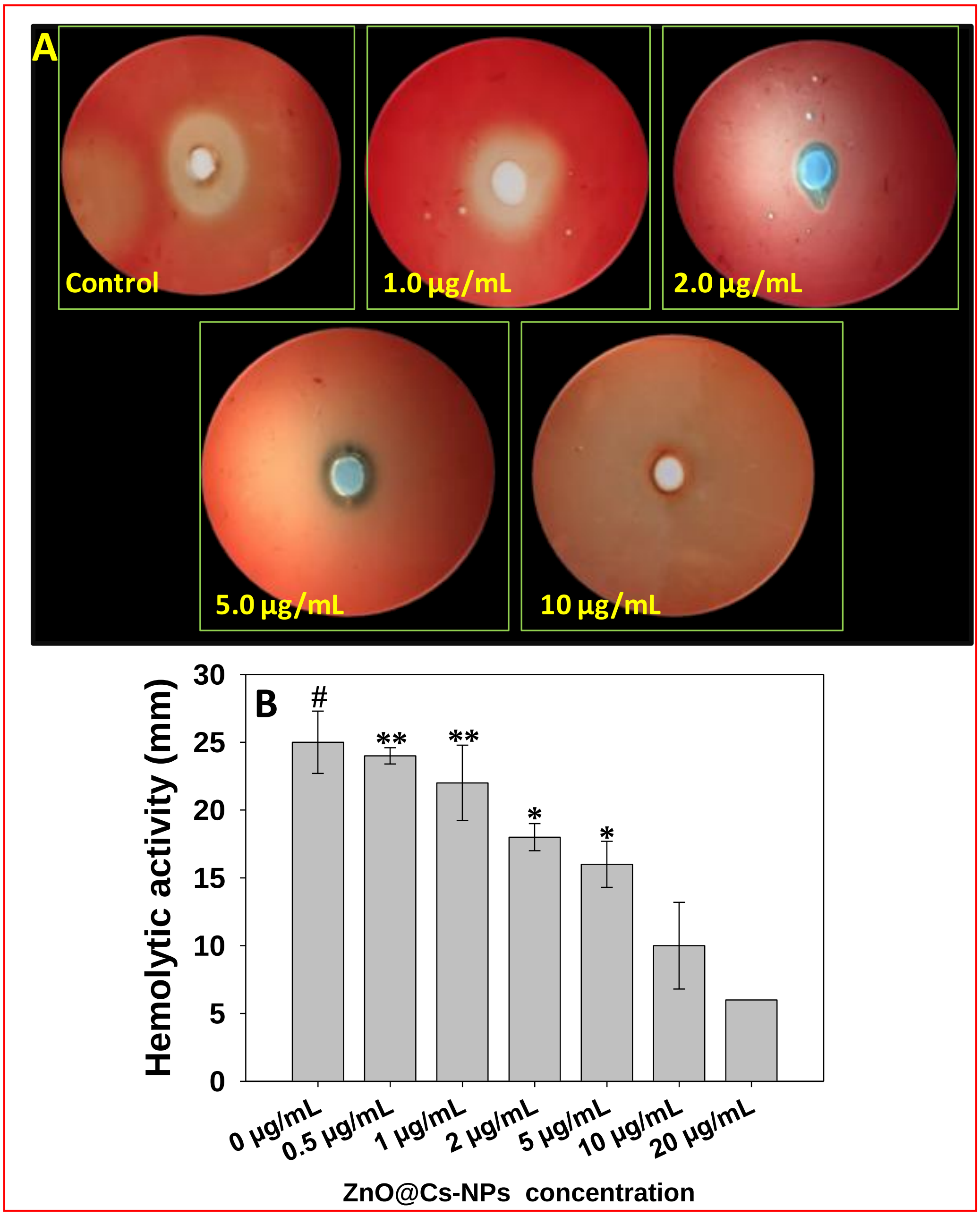

Figure S1: Impact of varying concentrations (0, 1, 2, 5 and 10 μgmL^{-1}) of green synthesized ZnO-NPs on qualitative estimation of hemolycin produced by *Pseudomonas aeruginosa* PAO1 (Panel A). Panel B represent the effect of ZnO@*Cs*-NPs on quantification of hemolysin production. In these figures, the histograms represent the mean value of three independent replicates (n = 3), while error bars show standard deviation (SD, n = 3) with a significance of *$p \leq 0.05$, **$p \leq 0.005$, and # $p \leq 0.001$. The symbols *, ** and # represent the significant difference between treatment with untreated control (i. e. 0 μg mL^{-1}).
